# Immune system development and age-dependent maintenance in Klotho-hypomorphic mice

**DOI:** 10.18632/aging.102121

**Published:** 2019-07-28

**Authors:** Wandi Sandra Zhu, Lynette Naler, Robert W. Maul, Michelle A. Sallin, Jyoti Misra Sen

**Affiliations:** 1 National Institute on Aging, National Institutes of Health, Baltimore, MD 21224, USA; 2 Department of Medicine, The Johns Hopkins University School of Medicine, Baltimore, MD 21287, USA; 3 Current address: Department of Immunology and Microbiology, University of California San Francisco, San Francisco, CA 94143, USA; 4 Current address: Department of Chemical Engineering, Virginia Tech, Blacksburg, VA 24061, USA

**Keywords:** aging, klotho hypomorphic allele, C57BL/6 background, immune cells, vitamin D3

## Abstract

Circulating Klotho peptide hormone has anti-aging activity and affects tissue maintenance. Hypomorphic mutant Klotho [*kl/kl*] mice on C57BL/6xC3H, BALB/c and 129 genetic backgrounds, show decreased Klotho expression that correlate with accelerated aging including pre-mature death due to abnormally high levels of serum vitamin D. These mice also show multiple impairments in the immune system. However, it remains unresolved if the defects in the immune system stem from decreased Klotho expression or high vitamin D levels in the serum. Transfer of the *kl/kl* allele to pure C57BL/6 genetic background [B6-*kl/kl*] significantly reduced expression of Klotho at all ages. Surprisingly, B6-*kl/kl* mice showed normalized serum vitamin D levels, amelioration of severe aging-related phenotypes and normal lifespan. This paper reports a detailed analysis of the immune system in B6*-kl/kl* mice in the absence of detrimental levels of serum vitamin D. Remarkably, the data reveal that in the absence of overt systemic stress, such as abnormally high vitamin D levels, reduced expression of Klotho does not have a major effect on the generation and maintenance of the immune system.

## Introduction

Aging-related changes in the immune system occur at two stages of life. The first begins at a young age in humans and mice, when the thymus begins to involute as the thymic micro-environment deteriorates, followed by reduction in the number of developing thymocytes. Additionally, and the bone marrow [BM] shows changes including an increase in the frequency of hematopoietic stem cells [HSCs] that provide short term re-population of immune cells and a decrease in the frequency of HSCs that provide long-term re-constitution [1, 2]. The second phase of immune system decline takes place in much older humans and mice and correlates with decreased ability to fight infections [3,4], inadequate response to vaccination [5–7] and decreased protection from cancer [8–10]. Despite thymic involution and BM changes, the peripheral immune system in adult mice remains reasonably stable until age-related decline is noted at a much older age. Stability of the immune system in adults is indispensable as immune cells play a critical role in tissue preservation and protection from infections and cancer [11]. However, mechanisms that regulate the maintenance of immune system during adult life remain to be adequately elucidated.

Klotho, a type I single-pass transmembrane protein with beta-glucuronidase activity, has been implicated in influencing the aging process [12]. While, the transmembrane protein is predominantly expressed in the kidney and choroid plexus of the brain, and not in immune cells [13–16], a soluble form of the Klotho peptide is found in blood, urine and cerebrospinal fluid and possesses hormone-like activity that can affect aging of tissues in which Klotho is not expressed [17]. Importantly, the levels of Klotho hormone decline with normal aging in mice and in humans [18], suggesting a role in immune system maintenance. A mutant *Klotho* allele [*kl/kl*], which reduces Klotho expression, was isolated in an unbiased mutagenesis scheme using C3HxC57BL/6 mice. The mutant mice displayed traits of premature aging and were short lived [19]. In contrast, overexpression of the gene extended the lifespan of mice, implicating the protein in aging-related functions [20]. Klotho deficiency in C3HxC57BL/6, BALB/c and 129 mice resulted in detrimental phenotypes in the immune system, including severe thymic atrophy and defective B cell lymphopoiesis [19,21,22]. In 129-Klotho-deficient mice, a significant increase in erythropoietin production by the kidney resulted in increased erythropoiesis in the BM and spleen and impaired hematopoiesis [23]. These studies show that Klotho-deficiency can affect the immune system, but the role of Klotho in the maintenance of immune system was difficult to assess in these systems due to dramatically accelerated aging and premature death at a young age.

Expression of *kl/kl* (Klotho- *Kl* *chr5:150,950,607-150,995,817)* in C3HxC57BL/6 mice leads to higher levels of minerals and active vitamin D in the serum, which correlates with susceptibility to age-related phenotypes and pre-mature death [12]. Surprisingly, transfer of the *kl/kl* allele to the pure C57BL/6 genetic background [*B6-kl/kl*] restored lifespan comparable to C57BL/6 mice [24]. Amelioration of aging-related phenotypes were associated with comparable levels of serum phosphate, calcium and active vitamin D levels in B6*-kl/kl* and C57BL/6 mice [25]. Remarkably, it was demonstrated that genetic variations [deletions and substitutions] in super enhancer-like regulatory regions at the *Cyp24a1* (*Cyp24a1* *chr2:170,480,957-170,499,145)* genetic locus in C3H, BALB/c and 129 mice resulted in lower basal levels of *Cyp24a1* expression in kidney compared to C57BL/6 mice. These data suggested that higher basal expression of *Cyp24a1* in C57BL/6 mice allowed proper metabolism of vitamin D in B6*-kl/kl* mice and rescued them from detrimental phenotypes that result from high levels of serum vitamin D [25]. Importantly, B6*-kl/kl* mice provide the opportunity to assess the effects of decreased expression of Klotho on immune cell development and maintenance of the immune system, without the confounding effects of high serum vitamin D and acute premature aging.

In this report, we demonstrate that immune cell development and age-dependent changes in the two primary immune compartments, bone marrow and the thymus, were comparable in age-matched B6*-kl/kl* and C57BL/6 mice. Remarkably, B6*-kl/kl* mice showed age-associated thymic involution and changes in the BM at a rate comparable to control C57BL/6 mice. Analysis of peripheral immune organs in adult mice showed that the frequency of immune cells in B6*-kl/kl* mice was comparable to age-matched C57BL/6 mice. Thus, these data reveal that all major immune cells developed normally and were maintained at similar levels in B6*-kl/kl* mice despite significantly low levels of Klotho expression. Overall, these data demonstrate a minor role of circulating Klotho in immune system development and maintenance in adult mice.

## RESULTS

### The decrease in Klotho expression results in lower body weight in the B6-kl/kl mice

Klotho-hypormorphic C57BL/6xC3H*-kl/kl* mice [19] were backcrossed to C57BL/6J mice for 10 generations (*B6-kl/kl*). The purity of the genetic B6*-kl/kl* background was confirmed by typing ten mice for 96 C57BL/6 microsatellite markers using DNA extracted from the tails. All mice were homozygous for at least 94 out of the 96 markers analyzed (background ‘purity’ >98.96%) (Table 1). These data demonstrate that B6*-kl/kl* mice were syngenic with C57BL/6 mice.

**Table 1:** Genotyping of 96 microsatellite markers specific for a C57BL/6 background.

**#** **(Sex)**	**1** **(F)**	**2** **(F)**	**3** **(F)**	**4** **(F)**	**5** **(F)**	**6** **(M)**	**7** **(M)**	**8** **(F)**	**9** **(F)**	**10** **(M)**	**(mean ± SEM)**
**non-C57BL/6 markers**	0	0	0	0	0	1	0	0	0	0	0.1 ± 0.10
**C57BL/6 heterozygous makers**	0	1	1	1	2	0	0	2	1	1	0.9 ± 0.23
**C57BL/6 homozygous makers**	96	95	95	95	94	95	96	94	95	95	95 ± 0.21
**C57BL/6** **background purity (%)**	100.00	99.48	99.48	99.48	98.96	98.96	100.00	98.96	99.48	99.48	**99.42 ± 0.12**

**Figure 1:**
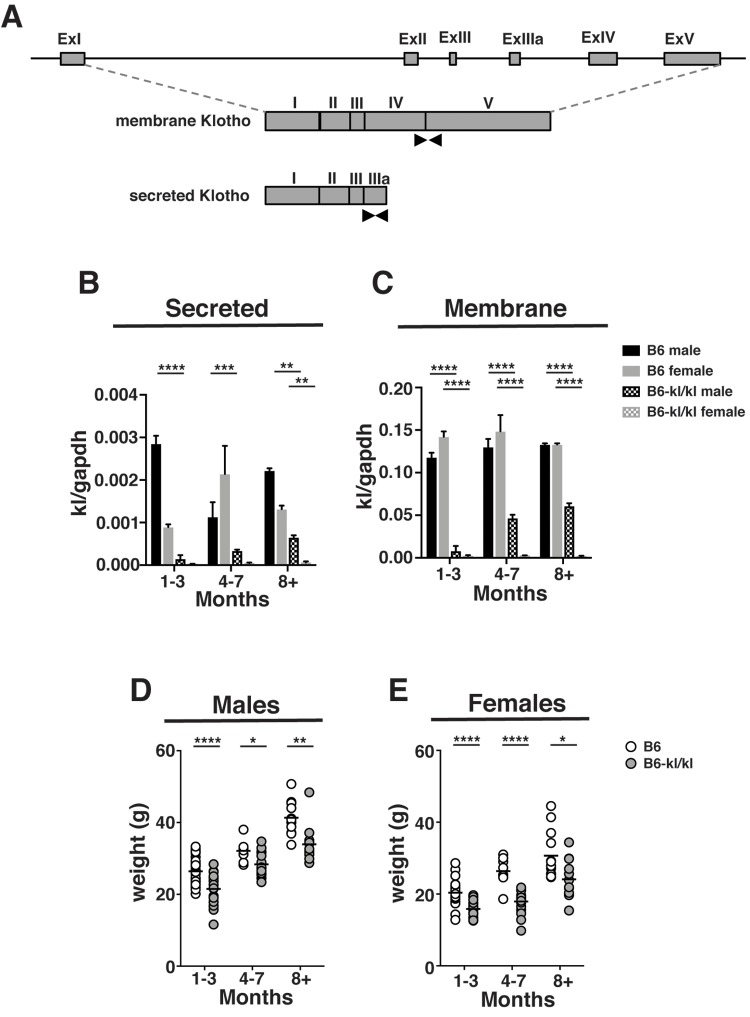
***B6-kl/kl* mice maintain an overall lower body weight than C57BL/6 as they age.** (**A**) A graphical representation of the *kl* gene. Black arrows indicate the primer sets used to amplify the secreted and membrane forms by quantitative PCR. (**B**) Secreted *kl* mRNA and (**C**) membrane *kl* mRNA expression in kidney normalized to *gapdh* at 1-3 months, 4-7 months, and 8+ months of age (n=2 per group). Bars represent standard error mean. Statistical significance determined by 2way ANOVA and Tukey’s multiple comparison test: ** p ≤ 0.01, *** p ≤ 0.001, and *** p ≤ 0.0001. Weights of C57BL/6 and *B6-kl/kl* (**D**) male and (**E**) female mice at 1-3 months, 4-7 months, and 8+ months of age. For male mice C57BL/6 1-3 months n=34, 4-7 months n=8, and 8^+^ months n=11 and *B6-kl/kl* 1-3 months n=21, 4-7 months n=14, and 8+ months n=11. For female mice C57BL/6 1-3 months n=38, 4-7 months n=8, and 8+ months n=13 and *B6-kl/kl* 1-3months n=17, 4-7 months n=19, and 8^+^ months n=19. Statistical significance determined by multiple t tests: * p ≤ 0.05, ** p ≤ 0.001, and **** p ≤ 0.0001.

In the kidney of mutant C57BL/6xC3H*-kl/kl* mice the level of *kl* mRNA expression was shown to be significantly decreased [19]. To confirm that our mice retained this feature on the C57BL/6 background, expression of secreted and membrane *kl* mRNAs in kidney were analyzed by qPCR (Figure 1A). The data show that B6*-kl/kl* mice express significantly lower levels of secreted and membrane forms of *kl* mRNA (Figure 1B and C). The immune system is likely to be most affected by the expression of the soluble form, which was also expressed at significantly lower levels in B6*-kl/kl* mice (Figure 1B). These data show that the hypomorphic allele [*kl/kl*] was appropriately transferred to the C57BL/6 background in B6-*kl/kl* mice.

The C57BL/6xC3H*-kl/kl* mice lived a short lifespan and had reproductive organ defects [19]. In this study, B6*-kl/kl* mice lived a lifespan that was indistinguishable from littermates that were not mutant, and showed no abnormality in reproductive organs or impaired reproductive capability (data not shown). The C57BL/6xC3H*-kl/kl* mice were significantly smaller in size [19]. Likewise, both male and female B6*-kl/kl* mice in this study showed significantly lower body weight (Figure 1D and E) as was previously reported [24]. These results show that the effects of decreased Klotho expression on lifespan and the premature aging-related phenotypes were largely alleviated on a C57BL/6 background.

### Frequency of immune cells in the Bone Marrow (BM) of B6-kl/kl mice was comparable to age-matched C57BL/6 mice

**Figure 2:**
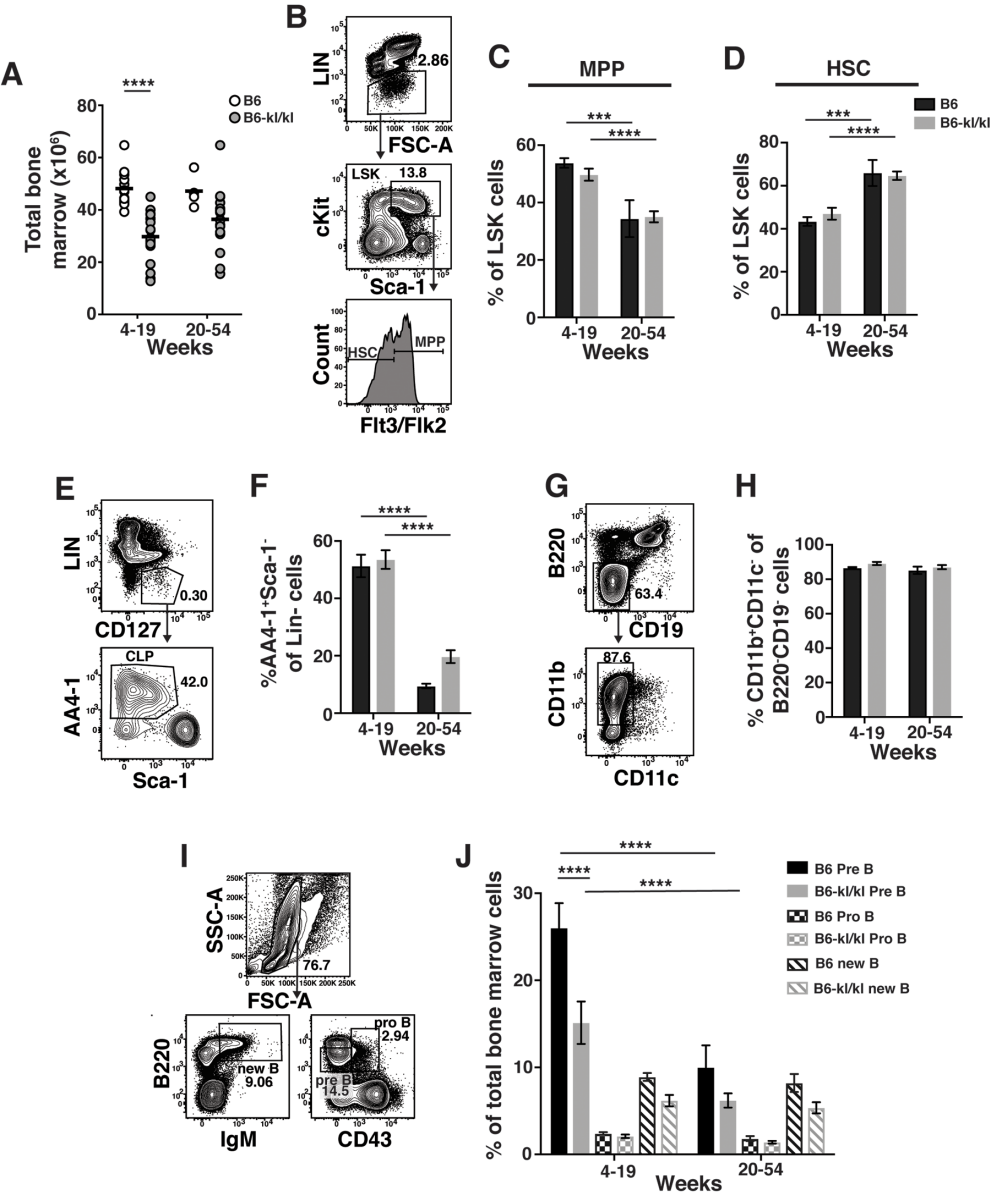
**Immune cells in the bone marrow have a similar composition in C57BL/6 and *B6-kl/kl* mice.** (**A**) Total bone marrow (BM) cells in C57BL/6 and *B6-kl/kl* at 4-19 weeks (C57BL/6 n=15 and *B6-kl/kl* n=18) of age or 20+ weeks (C57BL/6 n=4 and *B6-kl/kl* n=18) from pooled male and female mice. Statistical significance determined by multiple t tests: **** p ≤ 0.0001. (**B**) Representative flow cytometry plot and (**C**) frequency of MPP and (**D**) HSC of the LSK (Lin^-^cKit^+^Sca1^+^) cells. C57BL/6 n=15, 4-19 weeks; n=4, 20+ weeks. *B6-kl/kl* n=18, 4-19 weeks; n=18, 20+ weeks. Statistical significance determined by 2way ANOVA and Tukey’s multiple comparison test: *** p≤ 0.001 and **** p≤ 0.0001. (**E**) Representative flow cytometry plot of CLP (Lin^-^CD127^+^AA4.1^+^Sca1^low^), and (**F**) frequency of CLP in of Lin^-^CD127^+^ cells. C57BL/6 n=15, 4-19 weeks; n=4, 20+ weeks. *B6-kl/kl* n=18, 4-19 weeks; n=18, 20+ weeks. Statistical significance determined by 2way ANOVA and Tukey’s multiple comparison test: **** p ≤ 0.0001. (**G**) Representative flow cytometry plot of Myeloid cells (CD11b^+^CD11c^-^) and (**H**) frequency of CD11b^+^CD11c^-^ cells of B220^-^CD19^-^ cells. C57BL/6 n=10, 4-19 weeks; n=4, 20+ weeks. *B6-kl/kl* n=15, 4-19 weeks; n=15, 20+ weeks. (**I**) Representative flow cytometry plot for Pre B cells (B220^low^CD43^-^), Pro B cells (B220^+^CD43^+^) and new B cells (B220^+^IgM^+^) and (**J**) frequency of B cell subsets from BM. C57BL/6 n=15, 4-19 weeks; n=4, 20+ weeks. *B6-kl/kl* n=18, 4-19 weeks; n=16, 20+ weeks. Statistical significance determined by 2way ANOVA and Tukey’s multiple comparison test: **** p ≤ 0.0001.

We sought to determine the effect of Klotho on immune cell development and maintenance in adult mice. We divided adult mice into young-adult (young-4-19 weeks) and older-adult (old-20-54 weeks) group. Total number of BM cells recovered from B6*-kl/kl* mice were significantly reduced in young-adult and older-adult B6*-kl/kl* mice compared to age-matched C57BL/6 mice (Figure 2A). This reduction in cell number is most likely due to the overall smaller size of the B6*-kl/kl* mice (Figure 1B and C). As mice age the frequency of multi-potential progenitors (MPP) and short-term HSCs increase [2]. These age-related changes were not affected by decreased Klotho expression as B6*-kl/kl* mice did not show differences in the frequency of MPPs or HSCs compared to age-matched C57Bl/6 mice (Figure 2B, C and D). Importantly, the comparable age-associated loss of MPPs and gain of HSCs in both C57Bl/6 and B6*-kl/kl* mice indicate normal maintenance with age in adult mice. Common lymphoid progenitors (CLPs) were also present in similar frequencies in the B6*-kl/kl* mice compared to the C57Bl/6 mice and showed an analogous and significant loss with age (Figure 2E and F). B6*-kl/kl* mice showed comparable myeloid cells in the bone marrow compared to C57Bl/6 mice (Figure 2G and H). There was a reduction in the frequency of pre-B cells in the young B6*-kl/kl* mice, but this did not affect B cell development as B cells, pro B cells and new B cell frequencies were not reduced (Figure 2I and J). Overall, in the B6*-kl/kl* BM, immune cells developed and were maintained at comparable frequencies to age-matched C57BL/6 mice.

### T cell development, maintenance of thymic epithelial cells and age-related thymic involution in B6-kl/kl mice was comparable to age-matched C57BL/6 mice

**Figure 3:**
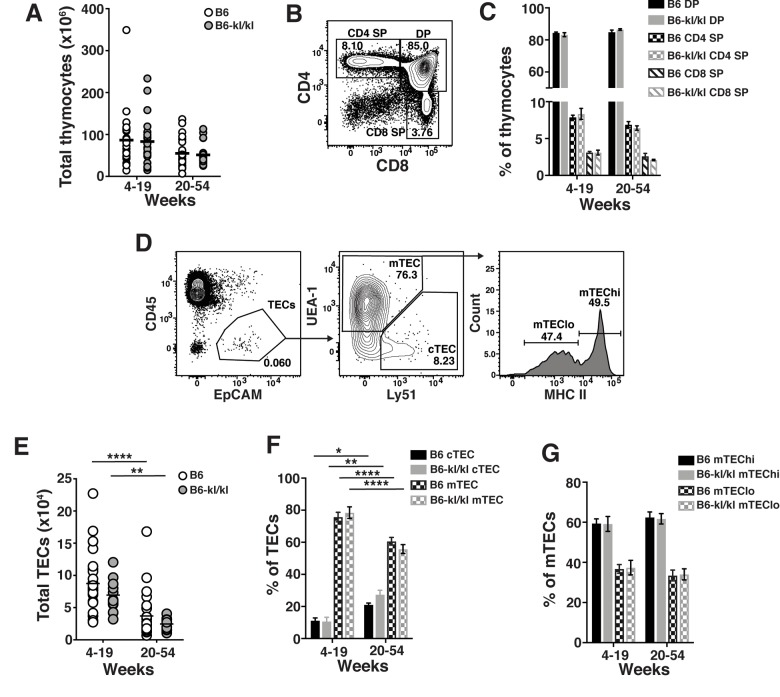
**C57BL/6 and *B6-kl/kl* mice have similar thymocyte and thymic epithelial cell (TEC) populations.** (**A**) Total thymocytes in C57BL/6 and *B6-kl/kl* at 4-19 weeks (C57BL/6 n=30 and *B6-kl/kl* n=22) of age or 20+ weeks (C57BL/6 n=25 and *B6-kl/kl* n=27) from pooled male and female mice. (**B**) Representative flow cytometry plot of thymocyte populations and (**C**) frequency of developing thymocyte populations based on CD4 and CD8 expression. C57BL/6 n=30, 4-19 weeks; n=25, 20+ weeks. *B6-kl/kl* n=19, 4-19 weeks; n=15, 20+ weeks. (**D**) Representative flow cytometry plots of cortical TEC (cTEC; CD45.2^-^EpCAM^+^Ly51^+^) and medullary TEC (mTEC; CD45.2^-^EpCAM^+^UEA-1^+^). (**E**) Total thymic epithelial cells (TECs) in C57BL/6 and B6-kl/kl at 4-19 weeks (C57BL/6 n=30 and *B6-kl/kl* n=22) of age or 20+ weeks (C57BL/6 n=25 and *B6-kl/kl* n=27) from pooled male and female mice. (**F**) Frequency of cTEC and mTEC of CD45.2^-^EpCAM^+^ cells. C57BL/6 n=27, 4-19 weeks; n=25, 20+ weeks. *B6-kl/kl* n=16, 4-19 weeks; n=15, 20+ weeks Statistical significance determined by 2way ANOVA and Tukey’s multiple comparison test: * p ≤ 0.05, ** p ≤ 0.01, and **** p ≤ 0.0001. (**G**) Frequency of hi and lo mTEC. C57BL/6 n=27, 4-19 weeks; n=25, 20+ weeks. *B6-kl/kl* n=16, 4-19 weeks; n=15, 20+ weeks.

To evaluate the effect of Klotho paucity during T cell development, thymocytes from B6*-kl/kl* mice at different age ranges were analyzed. Total thymocyte numbers in B6*-kl/kl* mice were comparable to age-matched C57BL/6 mice as were the frequencies of double positive (DP), single positive CD4 (CD4 SP) and single positive CD8 (CD8 SP) cells in the B6*-kl/kl* and C57BL/6 mice (Figure 3A-C). Thus, Klotho deficiency does not affect T cell development in the thymus.

Age-associated thymic involution is defined as an irreversible decrease in thymocyte numbers orchestrated by the decline in thymic epithelial cells (TECs) and the thymic environment [26]. To determine if TECs were affected by hypomorphic expression of Klotho in the C57BL/6 background, total numbers and subpopulations of TECs were analyzed and found to be comparable in age-matched B6-*kl/kl* and C57BL/6 mice (Figure 3D-G). Furthermore, all significant age-dependent changes noted in TECs were comparable in age-matched B6-*kl/kl* and C57BL/6 mice. Medullary TECs can be further differentiated into mTEC low (mTEClo) and mTEC high (mTEChi) based on expression level of major histocompatibility molecule II (MHCII). The frequency of these cells was also comparable between the two groups (Figure 3G). Together, this detailed analysis of the thymus shows that hypomorphic expression of Klotho did not impair T cell development and that age-related thymic atrophy occurred normally in B6*-kl/kl* mice compared to C57BL/6 mice.

### Innate and adaptive immune cell maintenance in the spleen of B6-kl/kl mice

**Figure 4:**
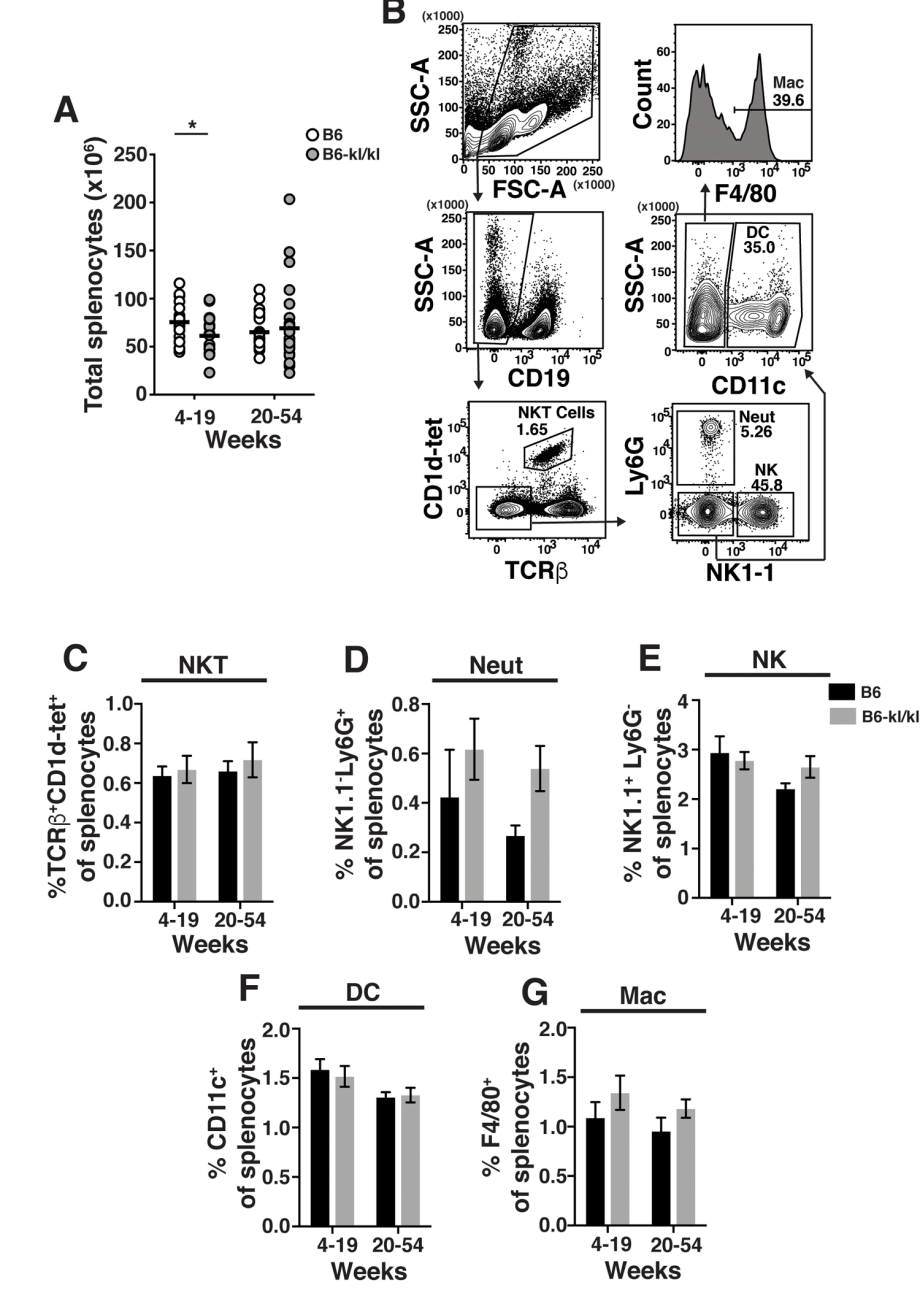
**Innate immune cell composition in the spleen is similar in C57BL/6 and *B6-kl/kl* mice.** (**A**) Total splenocytes in C57BL/6 and *B6-kl/kl* mice at 4-19 weeks (C57BL/6 n=25 and *B6-kl/kl* n=19) and 20+ weeks (C57BL/6 n=25 and *B6-kl/kl* n=27) of age from pooled male and female mice. Statistical significance determined by multiple t tests: * p ≤ 0.05. (**B**) Representative flow cytometry plots of NKT cells (CD19^-^CD1d^+^TCRβ^+^), Neutrophils (Neut) (CD19^-^CD1d^-^TCRβ-^-^NK1.1^-^Ly6G^+^), NK cells (CD19-CD1d^-^TCRβ^-^NK1.1^+^Ly6G^-^), dendritic cells (DC) (CD19^-^CD1d^-^TCRβ-NK1.1^-^Ly6G^-^CD11c^+^), and macrophages (Mac) (CD19^-^CD1d^-^TCRβ-NK1.1^-^Ly6G^-^CD11c^-^F4/80^+^). Frequency of (**C**) NKT, (**D**) neutrophils, (**E**) NK cells, (**F**) dendritic cells, and (**G**) macrophages. C57BL/6 n=13, 4-19 weeks; n=19, 20+ weeks. *B6-kl/kl* n=11, 4-19 weeks; n=16, 20^+^ weeks. Bars represent the standard error mean.

**Figure 5:**
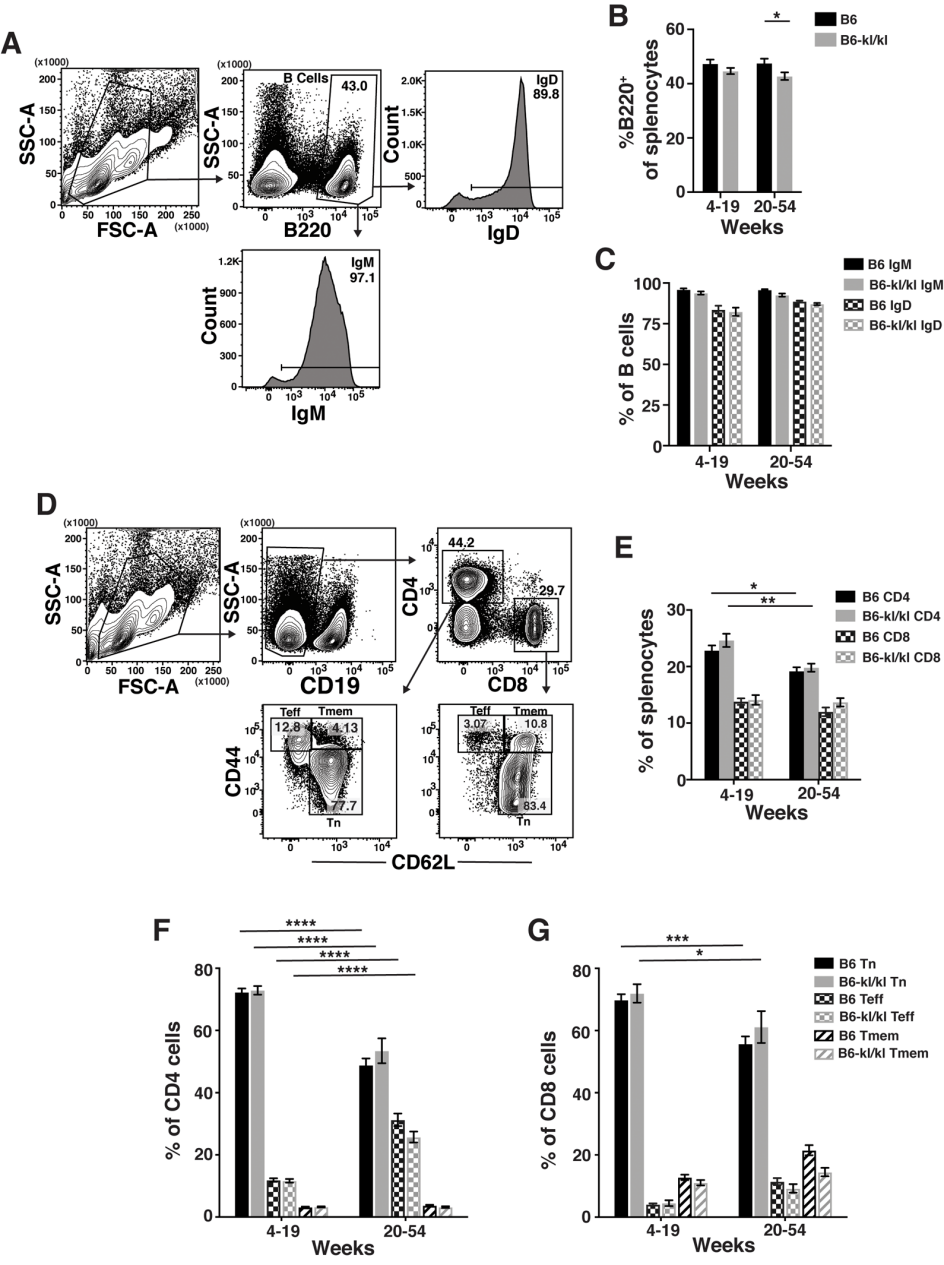
**Adaptive immune cell composition in the spleen is similar in C57BL/6 and *B6-kl/kl* mice.** (**A**) Representative flow cytometry plots of splenic B cells. Frequency of (**B**) total B cells and (**C**) IgM^+^ or IgD^+^ B cells in the spleen of C57BL/6 and B6-kl/kl mice at 4-19 weeks (C57BL/6 n=18 and B6-kl/kl n=18) and 20+ weeks (C57BL/6 n=16 and B6-kl/kl n=23) of age from pooled male and female mice. Bars represent the standard error mean. Statistical significance determined by 2way ANOVA and Tukey’s multiple comparison test: * p ≤ 0.05. (**D**) Representative flow cytometry plots of splenic naïve (Tn; CD62L^+^CD44^lo^), effector-phenotype (Teff; CD44^hi^CD62^lo^), and memory-phenotype (Tmem; CD44^hi^CD62^+^) CD4 T cells (CD19^-^CD4^+^) and CD8 T cells (CD19^-^CD8^+^). (**E**) Frequency of CD4 and CD8 T cells. C57BL/6 n=24, 4-19 weeks; n=25, 20+ weeks. *B6-kl/kl* n=19, 4-19 weeks; n=19, 20+ weeks. Statistical significance determined by 2way ANOVA and Tukey’s multiple comparison test: * p ≤ 0.05 and ** p ≤ 0.001. Bars represent the standard error mean. Frequency of naïve, effector-phenotype, and memory-phenotype of (**F**) CD4 T cells and (**G**) CD8 T cells. C57BL/6 n=24, 4-19 weeks; n=25, 20+ weeks. *B6-kl/kl* n=19, 4-19 weeks; n=19, 20+ weeks. Statistical significance determined by 2way ANOVA and Tukey’s multiple comparison test: *** p ≤ 0.001 and **** p ≤ 0.0001. Bars represent the standard error mean.

The spleens from B6*-kl/kl* mice were neither visibly abnormal nor hypercellular and demonstrated no enhanced erythropoiesis as noted in Klotho-deficient 129 mice [23] (Figure 4A). In the young B6*-kl/kl* there was a small reduction in the number of splenocytes compared to the C57Bl/6 mice, which was ameliorated with age and therefore did not represent a defect in maintenance of immune cells (Figure 4A). The overall assessment of the innate immune cells in the spleen included natural killer T cells (NKT), neutrophils (Neut), natural killer cells (NK), dendritic cells (DC), and macrophages (Mac) (Figure 4B). The frequency of these cell populations was comparable in the young-adult and older-adult B6*-kl/kl* mice compared to C57BL/6 mice (Figure 4C-G). There was a small but significant reduction in the frequency of B cells in the spleens of older-adult B6*-kl/kl* mice compared to C57BL/6 mice (Figure 5A and B), but no differences in frequency of IgD^+^ or IgM^+^ B cells (Figure 5C). B6*-kl/kl* had comparable frequency of naïve (Tn), effector-phenotype (Teff), and memory-phenotype (Tmem) T cells in both CD4 and CD8 T cell compartments compared to C57BL/6 mice (Figure 5D-G). Together these data demonstrate that adaptive and innate immune systems remained unaffected by decreased Klotho expression in the spleen of B6*-kl/kl* mice.

## DISCUSSION

In this report we demonstrate that the hormone Klotho does not control the development and maintenance of the immune system in adult mice. Specifically, *kl/kl* allele on a pure C57BL/6J background, after ten rounds of breeding with C57BL/6J mice, (B6*-kl/kl)* showed decreased expression of Klotho, but no age-related phenotypes or pre-mature death. Mutant animals showed no defects in fertility or lifespan but were smaller than age-matched C57BL/6 mice. Furthermore, the data overall demonstrate that in the absence of systemic stress from increased serum vitamin D [25], decreased expression of Klotho alone was not detrimental to immune cell development or maintenance of the immune system.

Aging-related effects of declining Klotho expression have been extensively documented. Since the identification of Klotho [19], scientists in several laboratories have defined alterations in mineral and vitamin D metabolic pathways through the interaction with FGF-23 that result in deleterious increase in serum phosphate, calcium and active vitamin D [27–30]. Accordingly, dietary and genetic manipulations that lowered serum levels of minerals and active vitamin D correlated with abrogation of aging-related phenotypes [31,32]. With respect to the regulation of serum vitamin D, recent analysis showed that higher basal expression of Cyp24a1 in C57BL/6 genetic background normalized active vitamin D levels in the serum of B6*-kl/kl* mice [25]. In the absence of increased vitamin-dependent systemic stress, B6*-kl/kl* mice showed no overt signs of distress and lived a life-span that was generally comparable to control C57BL/6 mice.

*kl/kl* allele on susceptible strains [C3H, BALB/c and 129], results in several acute effects on the immune system. The thymus is essentially absent in C3HxC57BL/6-*kl/kl* mice within a few weeks of birth [19] and deficits in B cell lymphopoieisis are noted after 2-weeks of age [22], when the detrimental effects of increased vitamin D levels in serum are the highest [33]. This suggests that the decrease in thymus size and B cell phenotype could be secondary to high vitamin D levels in the serum. Germline deletion of Klotho [on 129 genetic background] results in induction of erythropoietin (Epo) production in the kidney, which in turn induces abnormal generation of erythrocytes in the BM and subsequently in the spleen [23]. These phenotypes were not observed in B6*-kl/kl* mice. Unlike in the recently reported 129xC57BL/6-*kl/kl* mice [21], TEC populations and T cell development in B6-*kl/kl* mice thymus were also unaffected. These observations support the notion that the effects of Klotho-deficiency on the immune system could be attributed to systemic stress resulting from altered metabolic events, especially serum vitamin D levels. The observation that thymic involution and changes in the BM proceeded comparably in young-adult B6 and B6*-kl/kl* mice, taken with comparable presence of immune cells in the peripheral organs in young-adult and older-adult B6 and B6*-kl/kl* mice, suggest that immune system development and maintenance was normal in the absence of Klotho expression.

## MATERIALS AND METHODS

### Mice

Klotho-hypormorphic *kl/kl* mice on C3H/Jx C57BL/6J mixed background were provided by Dr. Makoto Kuro-o [19] and backcrossed for 10 generations to C57BL/6J mice obtained from The Jackson Laboratory to generate B6*-kl/kl* mice. B6*-kl/kl* mice were screened by conventional PCR immediately after weaning using the primer forward: 5’-tggagattggaagtggac-3’ together with the primers reverse: 5’-caaggaccagttcatcatcg-3’ and reverse: 5’-ttaaggactcctgcatctgc-3’ for the Klotho hypomorphic and for the WT genotype, respectively. All mice were bred and maintained in an animal facility at the National Institute of Aging (NIA) in a 12-hour light-dark cycle and fed ad libitum with a diet containing 0.7% phosphorus (0.4% non-phytate phosphorus). The studies were carried out in accordance with the recommendations in the Guide for the Care and Use of Laboratory Animals (NRC, 2010). The protocol was approved by the Animal Care and Use Committee of the NIA Intramural Research Program, NIH.

### C57BL/6 background genotyping for B6-kl/kl mice

Tail samples from B6*-kl/kl* mice were sent to the Animal Molecular Diagnostics Laboratory at Frederick National Laboratory for Cancer Research to verify congenicity with C57BL/6 strain. Briefly, 1cm tails from 10 mutant mice were digested and DNA extracted using DNeasy® Blood & Tissue Kit (Qiagen, Venlo, Netherlands). DNA samples were then quantified using a nanodrop (BMG Labtech, Offenberg, Germany) and screened for 96 C57BL/6 microsatellite markers distributed over 19 chromosomes using an ABI 3130 Genetic Analyzer.

### Tissue collection

Thymus, spleen and bone marrow were harvested and processed into single cell suspensions for flow cytometry analysis by following standard protocols. Thymic epithelial cells were obtained by gently teasing thymii to release the thymocytes and subsequently digested with 5mg/mL Liberase TH (Roche, Basel, Switerland) and 50mg/mL DNase I (Roche) at 37 °C, as previously described [34,35]. Bone marrow cells were harvested from the femur and tibia of the mice and processed accordingly to standard protocols.

### Flow cytometry analysis

Cells were incubated with Fc Block (CD16/32, BD Biosciences, San Jose, CA), stained with antibodies and then fixed with 2% paraformaldehyde. Samples were acquired on the FACS Canto II (BD Biosciences) and analyzed using FlowJo (TreeStar, Ashland, OR). Dead cells were excluded using the eBioscience Fixable Viability Dye eFluor® 506 (ThermoFisher Scientific, Waltham, MA). The following antibodies conjugated to biotin, FITC, PE, PerCP-Cy5.5, PE-Cy5, PE-Cy7, APC, APC-Cy7 or Pacific Blue were used (ThermoFisher Scientific, BD Biosciences, BioLegend, San Diego, CA): anti-CD4 (GK1.5), anti-CD8 (53-6.7), anti-TCR-β (H57-597), anti-CD19 (6D5), anti-CD44 (IM7), anti-B220 (RA3-6B2), anti-IgM (RMM-1), anti-IgD (11-26c.2a), anti-NK1.1 (PK136), anti-F4/80 (BM8), anti-Ly6G (1A8), anti-CD11c (HL3), anti-CD62L (MEL-14), anti-EpCAM (G8.8), anti-CD45.2 (104), anti-Ly51 (BP-1), anti-MHCII (M5/114.15.2), anti-UEA-1 (B-1065, Vector Labs), anti-Sca-1 (E13-161.7), anti-cKit (2B8), anti-Flt3/Flk2 (A2F10), anti-CD127 (A7R34), anti-AA4.1 (AA4.1), anti-CD43 (S7), anti-CD11b (M1/70), and Streptavidin. The LIN cocktail contained anti-NK1.1, anti-CD11b, anti-GR1 (RB6-8C5), anti-TER119 (TER-119), anti-CD3e (145-2C11), anti-CD19, and anti-B220. APC- conjugated mouse CD1d tetramers loaded with glycolipid PBS-57 (CD1d-tet) and an unloaded tetramer comprised of only the glycolipid PBS57 were obtained from the tetramer facility of the US National Institutes of Health.

### Gene expression analysis

Kidneys from C57BL/6 and *B6-kl/kl* mice were collected for RNA extraction and qPCR analysis. Briefly, kidneys were disrupted and homogenized using a TissueRuptor II, and RNA was extracted from lysates using the RNeasy mini kit (Qiagen). DNA was eliminated from the samples by incubating with DNase (Qiagen). First strand cDNA synthesis was performed by using 1μg of total RNA together with oligo(dT)_12–18_ and the Invitrogen SuperScript II Reverse Transcriptase (Thermo Fisher Scientific), accordingly to the manufacturer's instructions. Quantification of *klotho* mRNA expression was conducted using real-time qPCR performed on an Applied Biosystems ViiA™ 7 Real-Time PCR System (Thermo Fisher Scientific). Primers were designed to amplify specific amplicons of the membrane *kl* (F:5’-ggctctgaaagcctacgtgttg-3’; R:5’-gggagctgagcgatcactaagt-3’), secreted *kl* (F:5’- tgctggctttcctctaggtcat-3’; R:5’-ttaggcgttctgatgctgtca-3’) and *gapdh* (F:5’-gtcgtggagtctactggtgtc; R:5’cagaaggggcggagatgatg-3’) genes. Each cDNA sample was diluted 5-fold and then 5μL of dilutions added to 5pmol of each primer and SYBR Green Master (Roche). The cycling parameters were: 10 min at 95 °C, followed by 40 cycles of 15 s at 95 °C and 1 min at 60 °C. Quantification of gene expression was performed by the E^^− ΔCt^ method using *gapdh* as the normalizer gene (where E stands for primer amplification efficiency). Each sample was quantified in triplicate and primer amplification efficiencies were calculated and validated with the standard curves obtained through the amplification of cDNA serial dilution.

### Statistical analysis

Data is presented as mean ± standard error mean. Significance was determined using 2way ANOVA, Tukey’s multiple comparison tests, and multiple *t*-tests with GraphPad Prism 6.0 (GraphPad, San Diego, CA) software.
